# Case Report: Guillain-Barré Syndrome Characterized by Severe Headache Associated With Metabotropic Glutamate Receptor 5 Antibody

**DOI:** 10.3389/fimmu.2022.808131

**Published:** 2022-03-21

**Authors:** Weiqian Yan, Cheng Zhao, Hainan Zhang, Zhiping Hu, Chunyu Wang

**Affiliations:** ^1^ Department of Neurology, The Second Xiangya Hospital, Central South University, Changsha, China; ^2^ Department of Urology, Xiangya Hospital, Central South University, Changsha, China

**Keywords:** mGluR5 antibody, Guillain-Barré syndrome, limbic encephalitis, headache, Hodgkin’s lymphoma

## Abstract

Autoantibodies to metabotropic glutamate receptor 5 (mGluR5) are known to be the cause of autoimmune encephalitis, particularly limbic encephalitis, closely related to Hodgkin’s lymphoma (HL). The involvement of peripheral neuropathy is rarely reported. In our case, mGluR5 antibody was found in a Guillain-Barré syndrome (GBS) patient accompanied by severe headache but without neuropsychiatric manifestations or HL. Presenting with severe headache, the patient developed progressive bilateral limb weakness, areflexia, and cranial nerve involvement consisting of eye movement disorder, restricted mouth opening and chewing, bilateral facial paralysis and bulbar palsy. Cerebrospinal fluid (CSF) revealed elevated CSF protein level and normal cell count, known as “albumino-cytological dissociation”. Oligoclonal IgG bands were found in both the CSF and serum. Electrophysiological studies revealed symmetrical sensory and motor neuropathy with a mixture of axonal and demyelinating features. Brain and spinal cord magnetic resonance imaging (MRI), as well as the electroencephalogram, were normal. The mGluR5 antibody was positive in both serum and CSF with a Cell-Based Assay (CBA). The patient responded well to intravenous gammaglobulin therapy, correlated with a reduction of mGluR5 antibody titer from 1:30 to 1:10 in the serum. After 6 months, the patient recovered completely without any sign of recurrence or neoplasm. This first case of mGluR5 antibody-associated GBS accompanied by severe headache shows that mGluR5-associated disorders are not limited to manifestations of limbic encephalitis and HL.

## Introduction

As written by the pathologist Ian Carr in 1982, his daughter Jane lost memory gradually, with the final diagnosis of Hodgkin lymphoma (HL) (1). He argued that “a circulating neurotransmitter-like molecule produced by neoplasm” caused the brain disease. Later, Lancaster et al. and Mat et al. identified antibodies to mGluR5 as Carr’s neurotransmitter-like molecule in 3 patients with limbic encephalitis and HL (2, 3). In 2014, another patient with limbic encephalitis and antibodies against mGluR5 were described, with no tumor observed during a 17-month follow-up (4). In 2018, a retrospective study investigated the serum or cerebrospinal fluid (CSF) of 14,475 patients with various neurologic disorders (including viral encephalitis, autoimmune encephalitis, neurodegenerative diseases, etc.), and reported 7 additional patients with mGluR5-associated encephalitis, who developed a complex neuropsychiatric syndrome (5). There are various neuropsychiatric abnormalities, ranging from mood and personality changes to fatigue, involuntary movements, headaches, disorientation, and anterograde amnesia. In our case, mGluR5 antibody was found in a Guillain-Barré syndrome (GBS) patient accompanied by severe headache, while no neuropsychiatric manifestation and HL was reported. Our study revealed that mGluR5-associated disorders were not limited to limbic encephalitis and HL, thereby further expanding the phenotypic spectrum of mGluR5-associated disorders.

## Case Presentation

**Figure 1:**
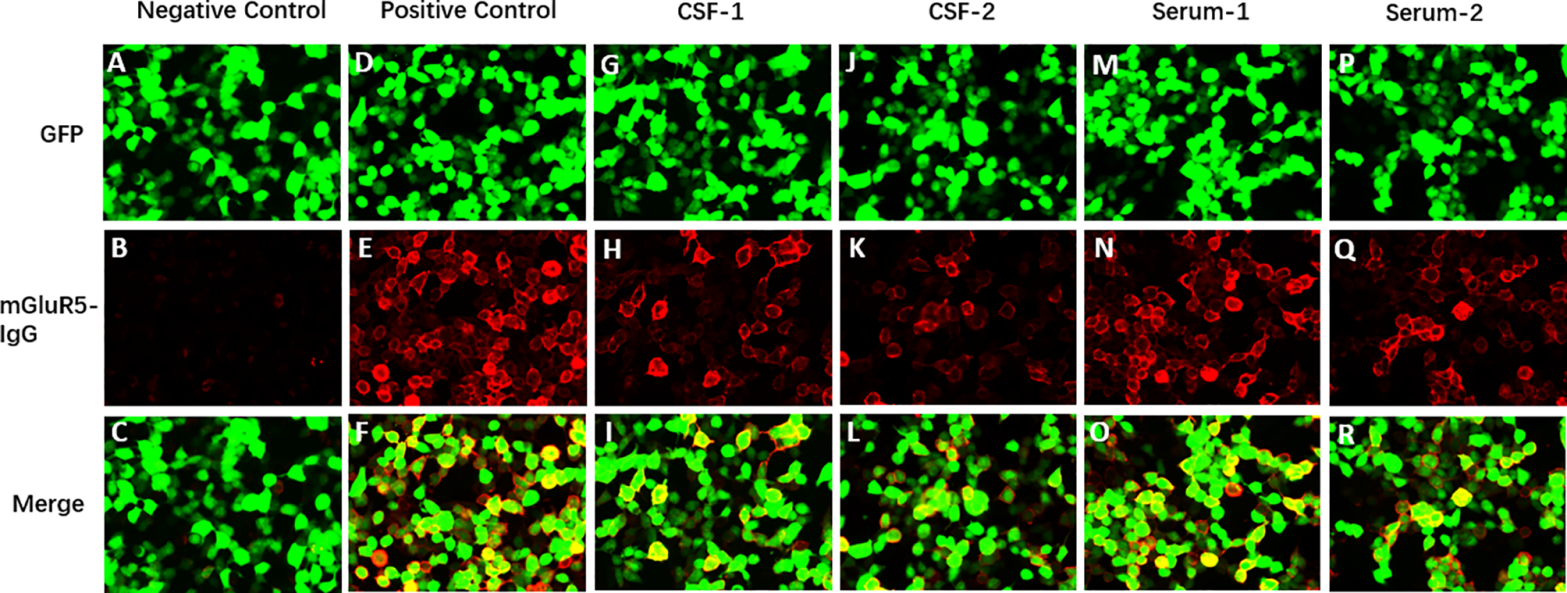
Immunofluorescence of anti-mGluR5 antibodies in the patient’s cerebrospinal fluid and serum. The anti-mGluR5 antibodies bound on the mGluR5 antigen expressed by the HEK293 cells and visualized by the immunofluorescence of fluorescein on the second antibody. **(A–F)** Cells transfected to express the target antigen (mGluR5) (green signal). **(G–L)** mGluR5 IgG antibodies of the patient bound to the cells (red signal). **(M-R)** Merged fluorescent images. **(A, G, M)** Example of mGluR5-antibody negative result. **(B, H, N)** Example of mGluR5-antibody positive result. **(C, I, O)** Fluorescent antibody staining for expression of mGluR5-antibody in the first CSF of the patient. **(D, J, P)** Fluorescent antibody staining for expression of mGluR5-antibody in the second CSF of the patient. **(E, K, Q)** Fluorescent antibody staining for expression of mGluR5-antibody in the first serum of the patient. **(F, L, R)** Fluorescent antibody staining for expression of mGluR5-antibody in the second serum of the patient.

A 44-year-old man with no prior headache history started to go through a serious bilateral swelling headache associated with nausea and vomiting followed by perioral herpes (the 1^st^ day), conventional painkillers failed to provide significant relief. The pain developed continuously, and severe back and limb pains began to develop with numbness in both distal ends of extremities. On the 2^nd^ day after initial symptoms, the patient started to have blurred vision, slurred speech, difficulty in opening the mouth, weakness in chewing, difficulty in swallowing (dysphagia), bitter taste on the tongue, choking on water, and difficulty in articulating words (dysarthria). On the 4^th^ day after initial symptoms, he consulted the neurology outpatient clinic in our hospital. He denied a history of cranial traumatisms, infectious diseases, or other precipitating factors. He was given a systematic neurological examination, revealing multiple cranial neuropathies, including bilateral III, IV, V, VI, VII, IX and X nerve involvement. Ocular fundi were normal. The muscle strength of his limbs was grade 4-5/5, and the muscle tone and tendon reflexes were normal. Hypoalgesia was not found in the distal extremities, and the remaining physical and neurological examination results were not significant. Notably, massive red rashes and flakes were noticed on both cheeks. On the 4^th^ day after initial symptoms (Admission Day), the lumbar puncture was performed, showing normal opening pressure (130 mm of water), elevated CSF protein level (1,627.00 mg/L), and normal cell count (0×10^6/L), which is a classical phenomenon called “alb——umino-cytological dissociation”. Oligoclonal IgG bands were found in both CSF and serum, along with negative CSF cytologic examination. IgG and IgM of ganglioside antibodies in serum and CSF including sulfatide, GM1, GM2, GM3, GM4, GD1a, GD1b, GD2, GD3, GQ1b, GT1a, and GT1b were negative, and CNS demyelinating antibodies (AQP4, MOG, GFAP, and MBP) and anti-neuronal antibody (amphiphysin, CV2/CRMP5, PNMA2 (Ma2/Ta), Ri, Yo, and Hu) were also negative. The Cell-Based Assay (CBA) test found that mGluR5 antibodies were positive in serum (1:30) and CSF (1:10) (**Figure 1**), while other autoimmune encephalitis antibodies were negative. We further looked for evidence of tumor and perfected PET-CT, which was negative.

On the 5^th^ day, the patient had further deterioration. His muscle strength of lower limbs weakened to grade 3-4/5, with the development of urinary retention. He revealed symmetrical decreased tendon reflexes in the extremities. The brain and spinal cord MRI with gadolinium showed no alteration or meningeal enhancement. There were no abnormalities in complete blood count, erythrocyte sedimentation rate, C-reactive protein, procalcitonin, creatine kinase, creatine kinase-MB, and glycosylated hemoglobin. Hepatitis B, Hepatitis C, HIV, and syphilis-related antibody tests were all negative. The thyroid function test result indicated thyrotropin 0.22uIU/ml↓. Serological antibody testing including ANA, ENA and c-/p-ANCA was negative. Electrophysiological studies revealed symmetrical demyelinating neuropathy with dysfunction of motor and sensory nerve fibers, indicated by reduced conduction velocities, prolonged distal motor latency, increased F-wave latency, reduced motor and sensory-evoked amplitudes, and abnormal temporal dispersion. (**Supplementary Material Table 1**). The patient was clinically diagnosed with acute inflammatory demyelinating polyneuropathy (AIDP) and initiated the treatment with a 5-day course of intravenous immunoglobulins (0.4g/kg/day). The patient’s condition became stable in the following weeks. His headache gradually improved and was easily controlled by common analgesics. On the 28^th^ day, a follow-up lumbar puncture was done, the CSF still showed an elevated protein level (1,147.00 mg/L) and normal cell counts, and the mGluR5 antibody titer of the serum declined to 1:10 (**Figure 1**), with no change in CSF titer levels.

During hospitalization, rehabilitation continuously contributed to recovering his muscle strength of limbs, and he could walk with support when discharged from the hospital (over 30 days post-admission). After being discharged from the hospital, the patient was not given sequential long-term immunosuppressive treatment. Also, he experienced complete resolution of symptoms without any sign of recurrence and tumors during a 6-months follow-up.

## Discussion

This is the first reported case of GBS associated with mGluR5 antibodies. Till now, only little mGluR5 associated encephalitis has been reported. All reported mGluR5-related disorders were summarized (**Supplementary Material Table 2**) to draw the following conclusions (2–6): (1) Most patients had a prodromal viral-like phase characterized by headache; (2) Most patients had HL or small cell lung cancer in combination; (3) Main clinical features were limbic encephalitis with neuropsychiatric abnormalities; (4) Brain MRI showed a wider involvement of brain structures beyond the limbic system; (5) CSF showed mild pleocytosis and normal protein level; (6) Improvement with immunotherapy and/or tumor treatment is common; (7) Neurologic relapse occurred in a small number of patients.

In our case, the patient developed progressive bilateral weakness and areflexia of legs and arms, which is the prerequisites for diagnosis of GBS (7, 8). Cranial nerve involvement included eye movement disorder, restricted mouth opening and chewing, bilateral facial paralysis and bulbar palsy. Short-term urinary retention indicated autonomic dysfunction. The changes in the electrophysiological and CSF studies were highly diagnostic in the recognition of GBS. The electrophysiological study is not required to diagnose GBS. However, Electrophysiological studies can differentiate between AIDP, AMAN, and AMSAN, which are the three electrophysiological subtypes of classical GBS. The result in our patient indicated typical AIDP (9). CSF examination was performed during the initial evaluation of our patient on admission day (the 4^th^ day after initial symptoms), the CSF revealed the classic finding in GBS known as “albumino-cytological dissociation” (the combination of elevated CSF protein level and normal CSF cell count) (8). The rapid progress of limbs weakness in the early stage was consistent with the albumino-cytological dissociation that showed up in the first week of the disease. A study in CSF of 474 GBS patients indicated that only 64% of patients have an increase of cerebrospinal fluid protein concentration, which highly depends on the timing of the lumbar puncture after weakness onset (49% on the first day to 88% after 2 weeks) (10). Our patient responded well to intravenous gammaglobulin therapy, correlated with a reduction of mGluR5 antibody titer from 1:30 to 1:10 within the serum, thereby indirectly demonstrating a link between mGluR5 antibodies and pathogenicity.

There are no reports of mGluR5 antibody-associated GBS, only two cases reported that anti-mGluR5 encephalitis patients with cranial nerve involvement besides manifestations of limbic encephalitis (5). Our patient showed no signs of typical limbic encephalitis, but only outstanding peripheral neuropathy and severe pain. Glutamate receptors (GluRs) serve as the major mediator of excitatory synaptic transmission within the central nervous system (CNS), which also distribute peripherally as an algogen and modulate nociceptor activities. GluRs are divided into ionotropic (iGluRs), or metabotropic (mGluRs) (11, 12). Glutamate plays its role through the mGluRs, kainate receptors, N-methyl-D-aspartate (NMDA) receptors, and 2-amino-3–(3-hydroxy-5-methyl-isoxazol-4-yl) propanoic acid (AMPA) receptors. Both the NMDA receptors and AMPA receptors are iGluRs. Antibodies against these two receptors bring out anti-NMDA receptor encephalitis and anti-AMPA receptor encephalitis, which could overlap with acute peripheral neuropathy (13). Conversely, antibodies against mGluRs lead to anti-mGluRs encephalitis. Theoretically, the mGluRs antibodies can also have an impact on not only the CNS but also the peripheral nervous system (PNS). However, the involvement of the PNS has rarely been emphasized. Studies have indicated that mouse models of peripheral neuropathy are also accompanied by changes in mGluR5 receptor levels (14, 15).

Severe headache, extremity, and back pain are outstanding manifestations in our case. Prospective studies have demonstrated that extremity and back pain is severe and highly prevalent throughout the spectrum of GBS, while the headache in the setting of GBS is rare (16). To our knowledge, only very few cases of typical Miller Fisher syndrome (MFS) with initial headache had been previously reported (17, 18). The mechanism of headache in GBS remains unknown, while there are several plausible explanations for the headache. Firstly, there is the impact of increased protein including CSF outflow obstruction at the level of arachnoid granulations. It is unlikely because the patient still had high levels of CSF protein when his headache was fully resolved. Secondly, serum autoantibodies activate the pathway of trigeminovascular pain, playing a role in headaches pathogenesis. Antibody-mediated damage of higher cervical nerves or trigeminal nerve roots acts as a peripheral generator of pain. Whether the effect of mGluR5 antibodies to glutamate receptors is related to the patient’s severe headache and back pain should be speculated. It is well known that glutamate plays an important role in pain sensation and transmission. For several decades, people have already explored the role of mGluRs within various pain forms (19). The novel subtype-selective pharmacological ligands of mGluRs have witnessed an increase of availability, which has made contributions to clarify the role of each mGluR subtype in pain processing (20).

## Conclusion

This is the first case of mGluR5 antibody-associated GBS patient accompanied by severe headache, which warns that mGluR5-associated disorders are not limited to manifestations of limbic encephalitis and Hodgkin’s lymphoma, therefore further expanding the phenotypic spectrum of mGluR5-associated disorders. Meanwhile, this article suggests that the scope of antibody detection for ganglioside antibody-negative GBS patients can be expanded, and more work should be done to clarify the relationship between mGluR5 antibody and GBS.

## Data Availability Statement

The original contributions presented in the study are included in the article/**Supplementary Material**. Further inquiries can be directed to the corresponding author.

## Ethics Statement

The studies involving human participants were reviewed and approved by the Medical Ethics Committee of the Second Xiangya Hospital of Central South University. The patients/participants provided their written informed consent to participate in this study. Written informed consent was obtained from the individual(s) for the publication of any potentially identifiable images or data included in this article.

## Author Contributions

WY and CW had full access to all the data in the study and take responsibility for the integrity of the data and the accuracy of the data analysis. HZ and ZH contributed equally to this work in administrative, technical, or material support. CZ was the joint author. All authors have read and agreed to the published version of the manuscript.

## Funding

This research is supported by the grant of National Natural Science Foundation of China (No. 81801123) to Weiqian Yan and Natural Science Foundation of Hunan Province (No. 2021JJ41055) to Cheng Zhao.

## Conflict of Interest

The authors declare that the research was conducted in the absence of any commercial or financial relationships that could be construed as a potential conflict of interest.

## Publisher’s Note

All claims expressed in this article are solely those of the authors and do not necessarily represent those of their affiliated organizations, or those of the publisher, the editors and the reviewers. Any product that may be evaluated in this article, or claim that may be made by its manufacturer, is not guaranteed or endorsed by the publisher.
