# Supplementary figures and images for: Case Report: Guillain-Barré Syndrome Characterized by Severe Headache Associated With Metabotropic Glutamate Receptor 5 Antibody
Source: Front Immunol. 2022 Mar 21;13:808131. doi: 10.3389/fimmu.2022.808131 (PMC8977415; doi:10.3389/fimmu.2022.808131)

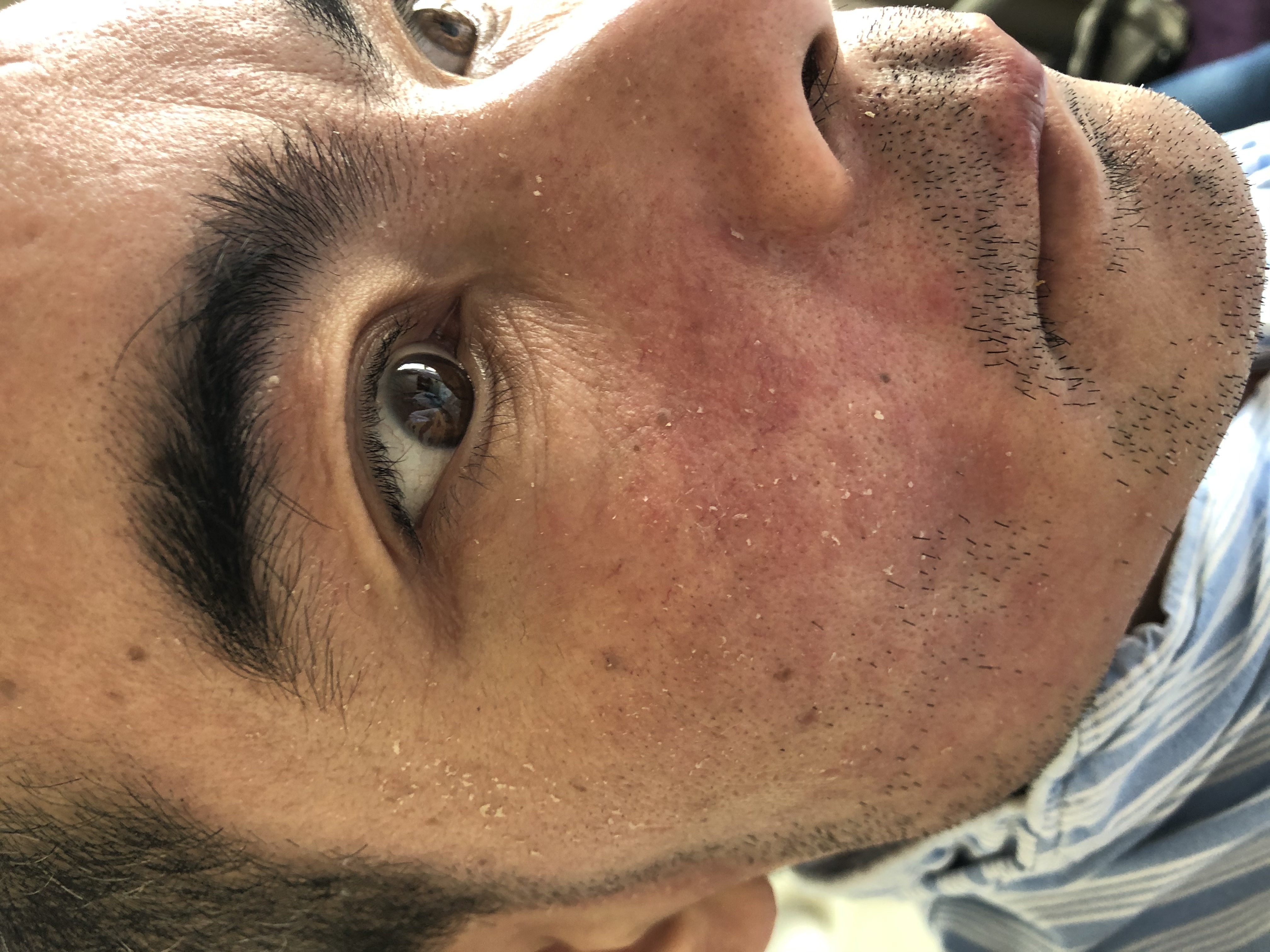

Supplement: Supplementary file 1 [file Image_1.jpeg]

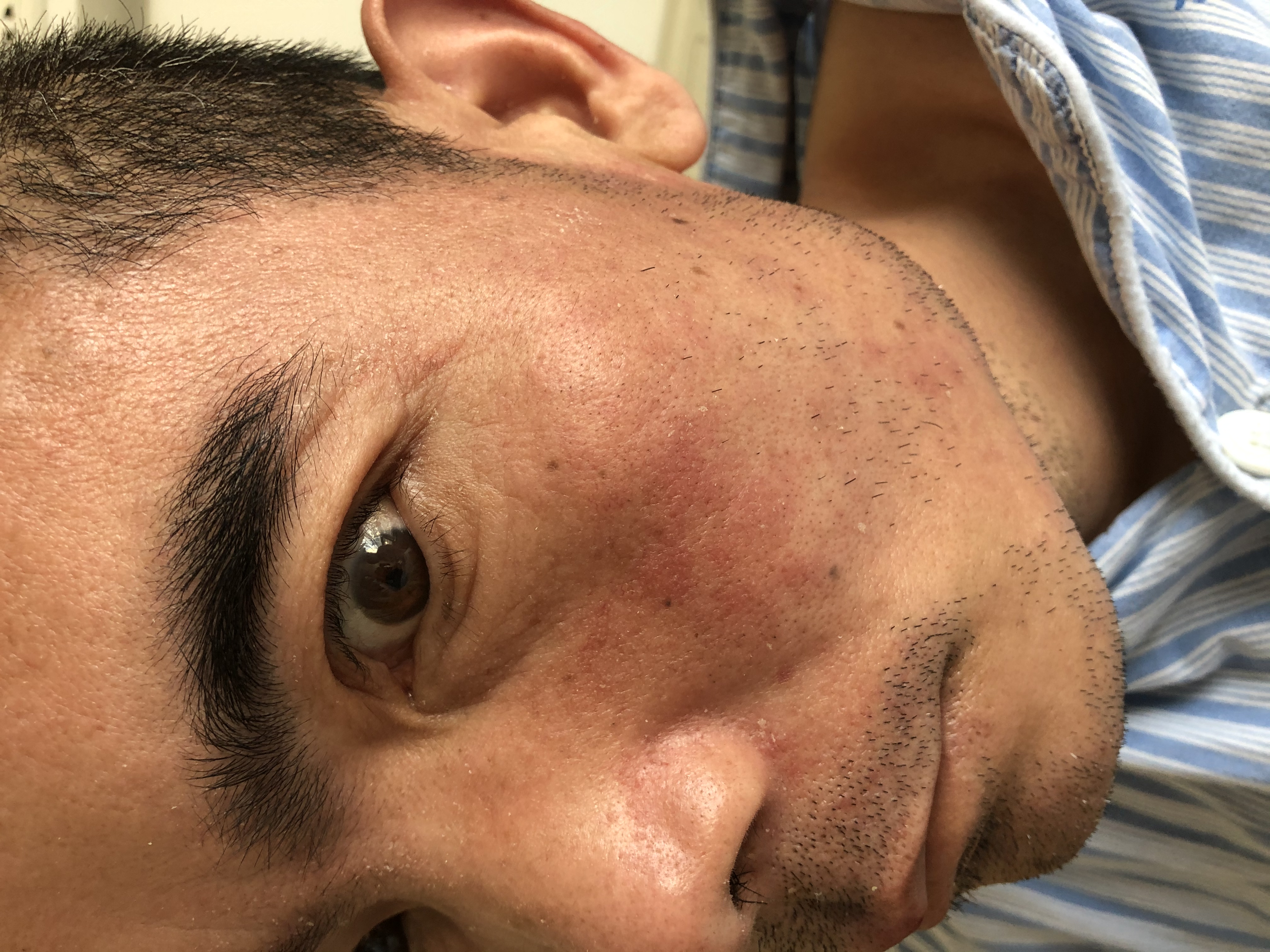

Supplement: Supplementary file 2 [file Image_2.jpeg]
