# Supplementary material for: Case Report: Guillain-Barré Syndrome Characterized by Severe Headache Associated With Metabotropic Glutamate Receptor 5 Antibody
Source: Front Immunol. 2022 Mar 21;13:808131. doi: 10.3389/fimmu.2022.808131 (PMC8977415; doi:10.3389/fimmu.2022.808131)
Supplement: Supplementary file 3 [file Table_1.docx]

Table 1 Serial nerve conduction studies and electromyography

| Time from onset | | Right | | Left | | **REF** | Right | | Left | | **REF** | Right | | Left | | **REF** |
| --- | --- | --- | --- | --- | --- | --- | --- | --- | --- | --- | --- | --- | --- | --- | --- | --- |
|  |  | D 1 | D 28 | D 1 | D 28 |  | D 1 | D 28 | D 1 | D 28 |  | D 1 | D 28 | D 1 | D 28 |  |
| **Sensory nerve conduction study** | | Onset latency (ms) | | | |  | Amplitude (mV) | | | |  | Conduction velocity (m/s) | | | |  |
| Ulnar | | 3.08 |  | 2.00 | 3.46 | ＜3.1 | 3.4 |  | 12.2 | 4.5 | ＞16 | 37.3 |  | 55.0 | 34.7 | ＞50 |
| Median | | 2.2 | 2.43 | 2.54 |  | ＜3.2 | 25.6 | 21.7 | 19.8 |  | ＞17 | 59.1 | 61.7 | 59.1 |  | ＞50 |
| Superficial peroneal | | 2.65 | 2.29 | 3.00 | 2.04 | ＜3.6 | 10.6 | 15.0 | 17.5 | 19.5 | ＞6 | 45.3 | 43.7 | 40.0 | 49.0 | ＞38 |
| Sural | | 1.95 | 1.90 | 1.77 | 2.00 | ＜3.6 | 41.8 | 38.2 | 41.4 | 37.8 | ＞6 | 48.7 | 52.6 | 53.7 | 70.0 | ＞38 |
| **Motor nerve conduction study** | | Onset latency (ms) | | | |  | Amplitude (mV) | | | |  | Conduction velocity (m/s) | | | |  |
| Ulnar | Wrist | 4.37 |  | 3.27 | 3.43 | ＜3.1 | 1.31 |  | 4.1 | 4.7 | ＞5 |  |  |  |  |  |
|  | Elbow | 8.21 |  | 6.81 | 7.38 |  | 0.96 |  | 4.2 | 3.4 |  | 59.9 |  | 56.5 | 58.2 | ＞50 |
| Media | Wrist | 5.04 | 6.08 | 4.06 |  | ＜3.9 | 1.58 | 1.25 | 2.3 |  | ＞4 |  |  |  |  |  |
|  | Elbow | 10.1 | 10.8 | 7.63 |  |  | 1.57 | 1.03 | 2.1 |  |  | 43.5 | 41.3 | 56.0 |  | ＞50 |
| Tibial | Ankle | 4.88 | 6.24 | 6.12 | 7.02 | ＜4.8 | 8.8 | 6.8 | 6.4 | 4.9 | ＞6 |  |  |  |  |  |
|  | Popliteal fossa | 13.4 | 13.0 | 14.5 | 15.1 |  | 7.9 | 5.5 | 6.0 | 3.8 |  | 44.6 | 51.8 | 47.7 | 43.3 | ＞40 |
| Deep peroneal | Ankle | 5.42 | 6.24 | 5.56 | 7.25 | ＜3.8 | 3.9 | 3.4 | 2.8 | 0.97 | ＞3 |  |  |  |  |  |
|  | fibular-head | 13.3 | 15.0 | 14.6 | 14.4 |  | 2.1 | 1.31 | 0.46 | 0.65 |  | 38.1 | 35.4 | 37.6 | 42.0 | ＞40 |
| **Electromyography** | | Fibrillations | | | |  | Positive sharp waves | | | |  | Recruitment | | | |  |
| Extensor digitorum m | | - |  | - |  |  | - |  | - |  |  | ↓ |  | ↓ |  |  |
| Abductor pollicis brevis m | | 0-1+ |  | 0-1+ |  |  | 0-1+ |  | 0-1+ |  |  | ↓ |  | ↓ |  |  |
| Abductor digiti minimi m | | 0-1+ |  |  |  |  | 0-1+ |  |  |  |  | ↓ |  |  |  |  |
| Biceps m | | - |  |  |  |  | - |  |  |  |  | ↓ |  |  |  |  |
| Iliopsoas m | | 0-1+ |  |  |  |  | 0-1+ |  |  |  |  | ↓ |  |  |  |  |
| Extensor digitorum brevis m | | 0-1+ |  |  |  |  | 0-1+ |  |  |  |  | ↓ |  |  |  |  |
| Tibialis Anterior m | | 0-1+ |  |  |  |  | 0-1+ |  |  |  |  | ↓ |  |  |  |  |
| Paraspinal m | | 1+ |  |  |  |  | 1+ |  |  |  |  | ↓ |  |  |  |  |
| Gastrocnemius m (lateral head) | | 0-1+ |  |  |  |  | 0-1+ |  |  |  |  | ↓ |  |  |  |  |
| Gastrocnemius m (medial head) | | 0-1+ |  |  |  |  | 0-1+ |  |  |  |  | ↓ |  |  |  |  |
| **Late responses** | | F-Latency (ms) | | | |  |  | | | |  |  | | | |  |
| Ulnar F-wave | | 21.1 |  | 28.3 |  | ＜30 |  |  |  |  |  |  |  |  |  |  |
| Median F-wave | | 32.1 |  | 20.8 |  | ＜29 |  |  |  |  |  |  |  |  |  |  |
| Tibial F-wave | | 37.1 |  | 36.5 |  | ＜55 |  |  |  |  |  |  |  |  |  |  |
| H-reflex | | NR |  | NR |  | ＜30 |  |  |  |  |  |  |  |  |  |  |

REF, referential normal value; D, Day; NR, no response.

The nerve conduction studies（NCS）revealed prolonged distal motor latency, decreased amplitudes of motor and sensory nerve conductions, and reduced conduction velocities, which suggest symmetrical demyelinating neuropathy of motor nerve fibers. The electromyography showed fibrillations and positive sharp waves with severely recruitment and normal unit potentials of tested muscles which suggest an active denervation of motor neuropathy. The results did not change significantly between Day 1 and Day 28.
