# Supplementary material for: Case Report: Guillain-Barré Syndrome Characterized by Severe Headache Associated With Metabotropic Glutamate Receptor 5 Antibody
Source: Front Immunol. 2022 Mar 21;13:808131. doi: 10.3389/fimmu.2022.808131 (PMC8977415; doi:10.3389/fimmu.2022.808131)
Supplement: Supplementary file 4 [file Table_2.docx]

Table 2 Systematic literature reviews of cases with mGluR5 antibodies

| **Ref** | **Age/**  **sex** | **Prodromal features** | **CNS**  **symptoms** | **PNS symptoms** | **signal**  **on MRI** | **CSF analysis** | **mGluR5 Ab** | **Tested antibodies** | **Tumor** | **Treatment** | **Follow up;**  **Prognosis** |
| --- | --- | --- | --- | --- | --- | --- | --- | --- | --- | --- | --- |
| 2 | 46/F | None | Depression, personality Change, seizure, short-term memory deficit, delusions, emotionally labile, myoclonic jerks | Normal | bilateral temporal, thalamus,  insula, frontal | 23 WBC, Protein 0.55 g/L | Serum：+  CSF：NA | Hu, Ri, Yo, CV2, Ma2, and amphiphysin, GAD | HL(IIIA) | AVBD chemotherapy,Steroids | 48 months, Complete recovery |
| 3 | 15/M | Headache, nausea | confusion, anxiety, extre-me agitation, auditory, visual hallucinations，generalized seizures | None | Bilateral post-erior parietal-occipital cortex | 114 WBC,  Protein 0.40 g/L | Serum：+  CSF：NA | Hu, Ri, Yo, CV2, Ma2, and amphiphysin, NMDAR | HL(IIA) | Radiotherapy | 72 months, Complete recovery |
| 3 | 35/M | Weight loss | Memory problems, personality changes, aggression | Right-sideⅩ, Ⅺ, Ⅻ cranial nerve palsies | upper pons. | 12 WBC,  Protein 0.87 g/L  OCB- | Serum: NA  CSF: + | GAD, LGI1, CASPR2 (VGKC), Hu, Ri, Yo, CV2, Ma2, and amphiphysin, 14-3-3 protein | HL(IIB) | AVBD chemotherapy | 35 months, Complete recovery |
| 4 | 30/F | Headaches, weight loss,  flu-like  symptoms | Personality, mood changes, memory problems, disorientation, affective incontinence, psychogenic nonepileptic seizures | None | Normal | 25 WBC, Normal protein,  IgG  index | Serum: +  CSF: + | NMDAR, AMPAR, GABA-BR, GABAAR, mGluR1, GlyR, LGI1, CASPR2, AQP4, DNER(Tr), GAD, Hu, Yo, Ri, Ma2, am-phiphysin, MAG, myelin | None | Steroids,  PE | 12 months, Partial |
| 5 | 75/M | Weight loss | Progressive ophthalmoplegia, postural hand tremor, gait instability, executive  dysfunction | None | Bilateral mesiotemporal lobes | 6 WBC,  increased IgG  index | Serum: +  CSF: + | NMDAR, AMPAR, GABA-BR, GABAAR, LGI1, CASPR2, DPPX, GlyR, GAD, IgLON5, D2R, neurexin-3α, and mGluR1, Hu, Ri, Yo, CV2, Ma2, and amphiphysin | SCLC | Steroids, IVIg,  Chemotherapy, Radiotherapy | 62 months, Partial |
| 5 | 40/F | Headache | Insomnia, anxiety, psychosis, auditory hallucinations, memory loss, dLOC, akinetic  mutism, orofacial dyskinesia | None | Normal | 45 WBC | Serum: +  CSF: NA | As above | None | Steroids, PE | 20 months, Complete recovery |
| 5 | 16/M | Headache | Psychosis, hallucinations,  poor sleep, dystonia,  generalized seizures, dLOC | None | Normal | 31 WBC, OCB | Serum: +  CSF: + | As above | HL(IIIB) | Steroids,  Chemotherapy, PE | 48 months, Complete recovery |
| 5 | 6/F | Rash, headache, flu-like  symptoms | Status epilepticus, dLOC,  aphasia, memory loss, poor sleep, dystonia  oculogyric crisis,  psychomotor slowness,  ataxia, speech and motor  regression, hypoventilation | None | Bilateral frontal (left > right)  and right occipital lobes,  cerebellum | 21 WBC, OCB- | Serum: NA  CSF: + | As above | None | Steroids, IVIg, RTX | 19 months, Partial |
| 5 | 20/F | Headache, flu-like  symptoms | Psychosis, emotional lability, thought disorder,  anterograde amnesia,  psychomotor slowing,  hypersomnia | None | Normal | 27 WBC, OCB | Serum：+  CSF：NA | As above | None | None | 96 months, Complete recovery |
| 5 | 15/M | None | developed altered behavior, memory loss, anxiety, irritability, visual  hallucinations, insomnia | Facial paralysis | Normal | 45 WBC, OCB | Serum: +  CSF: + | As above | HL(IA) | Steroids, IVIg,  Chemotherapy | 12 months,  Partial |
| 5 | 49/M | None | Insomnia, altered behavior, mania, emotional lability, psychomotor agitation, dLOC, seizures | None | Normal | 75 WBC, OCB | Serum: +  CSF: + | As above | None | Steroids | 5 months,  Partial |
| 6 | 68/M | night sweats, low grade fewer,  weight loss | depression, cognitive problems, disorientation,  inattention, psychomotor agitation, confusion, delusions of grandeur, auditory hallucinations, anterograde amnesia, psychomotor agitation | None | Normal | Normal WBC and protein, OCB | Serum: NA  CSF: + | NMDAR, AMPAR, VGKC, Hu, Ri, Yo, CV2, Ma2, and amphiphysin. AGNA-1  , ANNA-1,  2 and 3, CRMP-5  , PCA-1, 2, and 3 | HL(IIA) | Steroids | 1 months,  Partial |
|  | 44/M | Headache, nausea,  Perioral herpes, Rash, back and limb pains | None | Bilateral Ⅲ, Ⅳ, Ⅴ, Ⅵ, Ⅶ, and Ⅸ cranial nerve palsies, GBS, urinary retention | Normal | 0 WBC protein 1.63 g/L,  OCB | Serum: +  CSF: + | sulfatide, GM1, GM2, GM3, GM4, GD1a, GD1b, GD2, GD3, GQ1b, GT1a, and GT1b, AQP4, MOG, GFAP, MBP, Hu, Ri, Yo, CV2, Ma2, and amphiphysin, | None | IVIg | Partial |

M, male; F, female; NA, not available; IgG, immunoglobulin G; OCB, CSF oligoclonal bands; AVBD, chemotherapy with doxorubicin, vinblastine, bleomycin, and dacarbazine; HL, hodgkin's lymphoma; IVIg, intravenous immunoglobulin; PE, plasma exchange; RTX = rituximab; dLOC = decreased level of consciousness; mGluR, metabotropic glutamate receptor; GAD, glutamic acid decarboxylase; NMDAR, NMDA receptor; AMPAR, AMPA receptor; LGI1, leucine-rich glioma inactivated protein 1; CASPR2, contactin-associated protein-like 2; VGKC, voltage-gated potassium channel; GlyR, glycine receptor；AQP4, aquaporin-4; DNER, delta/notch-like epidermal growth factor; MAG, myelin Associated Glycoprotein; DPPX, dipeptidyl-peptidase-like protein 6; IgLON5, IgLON Family Member 5; D2R, dopamine2 receptor; AGNA, anti-Glial Nuclear antibody; ANNA, anti-neuronal nuclear antibody; CRMP-5, collapsin response-mediator protein-5; PCA, Purkinje Cell Cytoplasmic antibody; GM, monosialic ganglioside; MOG, myelin oligodendrocyte Glycoprotein; GFAP, glial fibrillary acidic protein; MAP, myelin basic protein.
